# Low-Dose Radiotherapy Ameliorates Advanced Arthritis in hTNF-α tg Mice by Particularly Positively Impacting on Bone Metabolism

**DOI:** 10.3389/fimmu.2018.01834

**Published:** 2018-09-18

**Authors:** Lisa Deloch, Anja Derer, Axel J. Hueber, Martin Herrmann, Georg Andreas Schett, Jens Wölfelschneider, Jonas Hahn, Paul-Friedrich Rühle, Willi Stillkrieg, Jana Fuchs, Rainer Fietkau, Benjamin Frey, Udo S. Gaipl

**Affiliations:** ^1^Department of Radiation Oncology, Universitätsklinikum Erlangen, Friedrich-Alexander-Universität Erlangen-Nürnberg (FAU), Erlangen, Germany; ^2^Department of Internal Medicine 3 and Institute for Clinical Immunology, Friedrich-Alexander-University Erlangen-Nürnberg (FAU) and Universitätsklinikum, Erlangen, Germany

**Keywords:** rheumatoid arthritis, inflammation, low-dose radiotherapy, bone metabolism, synovial-like fibroblasts, osteoclasts, osteoblasts

## Abstract

Inflammation and bone erosion are central in rheumatoid arthritis (RA). Even though effective medications for control and treatment of RA are available, remission is only seen in a subset of patients. Treatment with low-dose radiotherapy (LD-RT) which has been already successfully used for amelioration of symptoms in benign diseases should be a promising approach to reduce pain, inflammation, and particularly bone erosion in patients with RA. Even though anti-inflammatory effects of LD-RT are already described with non-linear dose response relationships, and pain-reducing effects have been clinically observed, the underlying mechanisms are widely unknown. Besides immune cells many other cell types, such as fibroblast-like synoviocytes (FLS), osteoclasts, and osteoblast are present in the affected joint and might be modulated by LD-RT. For this study, these cell types were obtained from human tumor necrosis factor-α transgenic (h*TNF-*α tg) mice and were consecutively exposed to different doses of ionizing radiation (0.1, 0.5, 1.0, and 2.0 Gy, respectively) *in vitro*. In order to study the *in vivo* effects of LD-RT within the arthritic joint, hind paws of arthritic h*TNF-*α tg mice were locally irradiated with 0.5 Gy, a single dose per fraction that is known for good clinical responses. Starting at a dose of 0.5 Gy, proliferation of FLS was reduced and apoptosis significantly enhanced with no changes in necrosis. Further, expression of RANK-L was slightly reduced following irradiation with particularly 0.5 Gy. Starting from 0.5 Gy, the numbers of differentiated osteoclasts were significantly reduced, and a lower bone resorbing activity of treated osteoclasts was also observed, as monitored *via* pit formation and Cross Laps presence. LD-RT had further a positive effect on osteoblast-induced mineralization in a discontinuous dose response relationship with 0.5 Gy being most efficient. An increase of the gene expression ratio of OPG/RANK-L at 0.1 and 0.5 Gy and of production of OPG at 0.5 and 1.0 Gy was observed. *In vivo*, LD-RT resulted in less severe arthritis in arthritic h*TNF-*α tg mice and in significant reduction of inflammatory and erosive area with reduced osteoclasts and neutrophils. Locally applied LD-RT can, therefore, induce a beneficial micro-environment within arthritic joints by predominantly positively impacting on bone metabolism.

## Introduction

Rheumatoid arthritis (RA) is an autoimmune disease that is characterized by chronic inflammation of the joints accompanied by the infiltration of activated immune cells, resulting in a progressive destruction of cartilage and bone (1, 2). Due to its relatively high prevalence as well as its severity RA is associated with high personal, social, and economic costs (3). During RA joints are destroyed, which manifests in joint swelling, tenderness, and ultimately destruction. This process is mediated by osteoclasts (OC), chondrocytes, and fibroblast-like synoviocytes (FLS). While OCs are responsible for the destruction of bone, enzymes such as matrix metalloproteinases (MMPs) that are secreted by neutrophils, chondrocytes, and FLS also degrade cartilage (4–7). FLS are considered to be the leading cells in joint destruction and strongly contribute to disease initiation, progression, and inflammation in RA (1, 8). FLS hyperplasia in particular is a crucial factor contributing to the severity of RA *via* the maintenance of local inflammation alongside with cartilage destruction (9). Neutrophils on the other hand are known to be involved in the initiation and maintenance of inflammation in RA. In addition, they are also involved in bone remodeling in RA patients (10, 11). Further, enhanced neutrophil extracellular trap (NET) activity has been correlated with systemic inflammatory markers (12).

Even though there are efficient anti-rheumatic treatment regimen available (2), there are still patients that do not respond completely (13); this applies in particular for patients with initial high disease activity or early joint damage (2), suggesting a need for additional therapeutic options (2, 13). For these patients, treatment with low-dose radiotherapy (LD-RT) might be beneficial to attenuate inflammation and bone destruction. Several clinical studies have already shown that LD-RT is effective for the treatment of degenerative musculoskeletal diseases and hyper-proliferative benign diseases (14–18).

Shortly after the discovery of X-rays, they were already used to treat RA (19) and in the 1970s X-ray therapy was considered to be the most effective physical-therapeutic approach in diseases that affect the spine and/or the joints (20). Today, LD-RT is used for many benign diseases such as painful degenerative skeletal disorders (17) while the application for RA is rising again. In the clinic, a single dose per fraction of 1.0 or 0.5 Gy (Gy) is widely accepted (17). However, a single dose of 0.5 Gy has been shown to be at least as effective as 1.0 Gy in terms of pain reduction in degenerative musculoskeletal disorders (21, 22). Even though the indications for the use of LD-RT are apparent, skepticism does still exist (23). This is mainly owed to the fear of radiation *per se*, but also to the fact that the mechanisms of attenuation of inflammation and delaying of bone destruction by LD-RT are not fully understood.

Low doses (≤1 Gy) of ionizing radiation are known to ameliorate inflammation based on a plethora of molecular modulations (24). These effects were predominantly examined *in vitro* and include, but are not limited to, induction of apoptosis in immune cells, reduction in leukocyte adhesion, reduced functionality of macrophages alongside altered transmigration, and chemotaxis of activated macrophages, the induction of anti-inflammatory cytokines, and alteration of the secretion of chemokines and cytokines (24–26). Therefore, radiation in the low and intermediate dose range can be regarded as radioimmunotherapy for inflammatory diseases. Of note is that in this dose range, responses do not follow a linear dose-effect relationship, but rather a biphasic curve where 0.5 Gy is often the most effective dose (27–30). These discontinuous dose dependencies are nowadays widely accepted (24). Additionally, investigations in various animal models of local inflammation with LD-RT were carried out and suggest a positive impact of LD-RT in the amelioration of symptoms (15, 31–34). Furthermore, advantageous effects mediated by LD-RT have also been shown in fracture healing (35).

However, the underlying mechanisms by which LD-RT may inhibit arthritis are still widely unknown as most experiments utilize local rather than systemic models of inflammation and the effects are often examined during acute rather than chronic inflammation. Furthermore, irradiation set-ups often do not resemble the clinical approach, as a local irradiation in small animal models is still a challenge.

We, therefore, aimed to investigate the effects of locally delivered LD-RT in the h*TNF-*α tg model of systemic inflammation that closely mimics human RA. We developed an irradiation set-up that allows for a precise local irradiation of the joints closely resembling the clinical situation. *Ex vivo* analyses of FLS, OCs, and osteoblasts cultures derived from arthritic mice receiving LD-RT were performed, as these cells are mainly involved in inflammation and degenerative processes in the joints. Since LD-RT should have an impact on multiple cells in the joints, consequently multiple modes of action and no single mechanism is expected to result in the final amelioration of symptoms.

## Materials and Methods

### Mice Treatment and Clinical Evaluation

Mice were maintained in a SPF facility under sterile atmosphere at the animal facility of the Universitätsklinikum Erlangen (Franz-Penzoldt-Center). The animal procedures have been approved by the “Regierung of Unterfranken” and were conducted in accordance with the guidelines of Federation of European Laboratory Animal Science Associations (FELASA). Heterozygous human TNF-α transgenic mice (strand Tg197) (36) were kindly provided by Prof. George Kollias (Fleming Institute, Vari, Greece). For irradiation purposes mice were kept under anesthesia and placed in an irradiation chamber made from led with an opening that allowed for precise irradiation of the left hind leg only. Irradiation with a single dose of 0.5 Gy was carried out using the Siemens Stabilipan 2 Orthovoltage (250 keV, 15 mA) equipped with a 1 mm copper filter, thus closely mimicking patient irradiation in the clinic. Clinical evaluation of the mice was carried out in a blinded manner as described previously (37).

### Histology and Histomorphometry

Hind paws of treated animals were fixed in 4% PFA (Sigma-Aldrich, Munich, Germany) and de-calcified in EDTA buffer. Paraffin sections (1 µm) from the specimen were stained using hematoxylin and eosin (H&E) in order to evaluate synovial inflammation as well as 1% toluidine blue (TB) for the assessment of proteoglycan loss. Osteoclasts and bone erosions were detected using tartrate-resistant acid phosphatase (TRAP) stain (Sigma-Aldrich, Munich, Germany). All slides were quantified using the OsteoMeasure™ Software (OsteoMetrics, Decatur, GA, USA), as described elsewhere (38).

Immunofluorescence staining of NET formation was carried out by staining paw slides with antibodies against elastase (Abcam, Cambridge, UK; 1:200), while DNA was stained using sytox™ Green, as previously described in Ref. (39). Images were taken with a Zeiss microscope at 20× magnification.

### *Ex Vivo* Irradiation Procedure

Irradiation of *ex vivo* cell cultures was performed using an Isovolt Titan X-ray tube (120 kV, 22.7 mA, variable time, GE Inspection Technologies, Hürth, Germany) with a 0.5 mm copper filter and a 5 cm Plexiglas^®^ plate. 24 h after plating, cells were irradiated with a single dose of X-rays (0.1, 0.5, 1.0, and 2.0 Gy).

### Fibroblast-Like Synoviocytes (FLS) Cultures

Fibroblast-like synoviocytes cultures were prepared in accordance with the protocol provided by Armaka et al. (40). Briefly, hind paws of hTNF-α tg mice were skinned and incubated for 4 h in a 1% collagenase type IV solution (Gibco, Carlsbad, CA, USA) at 37°C with shaking (1,400 rpm). Cells then were pooled and kept at standard culture conditions (37°C, 5% CO_2_; 95% humidity) in a medium containing 50% Dulbecco’s modified Eagle medium (DMEM; Pan Biotech, Aidenbach, Germany) and 50% F-12 medium (Gibco) supplemented with 10% fetal bovine serum (FBS; Biochrom, Berlin, Germany), 1% penicillin/streptomycin (PS; Gibco), and 1% low serum growth supplement (LSGS; Gibco). Cell purity was analyzed using flow cytometry. CD11b−, CD54+, and CD106+ cells were considered to be FLS. In total, five-independent FLS pools at P5 were used. Experiments were carried out by seeding 25,000 cells at least in triplicates in 6-well plate for the evaluation of cell growth, RNA isolation, and enzyme-linked immunosorbent assays (ELISAs) analysis or in T25 cell culture flasks for cell death analysis. 48 and 96 h after irradiation, respectively, samples were taken and processed.

### Osteoclast Culture

Bone marrow-derived osteoclasts (OCs) were isolated from the long bones of h*TNF-*α tg mice and kept in modified Eagle’s medium type α (α-MEM; Gibco) supplemented with 10% FBS (Sigma-Aldrich, Darmstadt, Germany), 1% PS (Gibco) at 37°C, and 5% CO_2_ overnight. Differentiation was carried out by adding 10 ng/ml M-CSF (PeproTech, Rocky Hill, NJ, USA) and 50 ng/ml RANK-L (PeproTech) to the culture medium. Cells were seeded at a density of 1 Mio cells/well in 24-well plate for RNA analysis and 500,000 cells/well in 48-well plate for TRAP stain. OC differentiation was evaluated *via* TRAP stain using a leukocyte acid phosphatase kit (Sigma-Adrich) according to the manufacturer’s instructions. TRAP positive cells with three or more nuclei were counted as osteoclasts.

### Osteoclast MTT Cell Proliferation Assay

MTT Assay (Trevigen^®^) for osteoclast cultures was used to determine cell viability; the assay was carried out following the manufacturer’s instructions. Briefly, cells were seeded in triplicates in 96-well plate and treated 1 day later with X-rays as described in Section “*Ex Vivo* Irradiation Procedure.” At the end of the differentiation process, MTT reagent was added and cells were incubated for 3 h. After the incubation time, MTT detergent was added and cells were kept at room temperature for 2 h in the dark before absorbance was measured at oD570-670.

### Osteoclast Pit-Formation Assay

In order to determine osteoclast activity/functionality, bone marrow cells were seeded onto cortical bovine bone slices (http://boneslices.com) (96-well format, 1 Mio cells/well) and cultured for 14 days as described in Section “Osteoclast Culture.” 24 h after seeding, cells were irradiated as described in Section “*Ex Vivo* Irradiation Procedure.” At the end of the cultivation process, cells were detached from bone slices using 0.25 M ammonium hydroxide for 5 min, followed by washing in PBS and staining using 1% TB. Bone slices were then scanned using an Epson Perfection 500V Photo scanner and analyzed using ImageJ.

### Osteoblast Culture

For osteoblast cultures mesenchymal cells were isolated from the calvariae of 3–6 days old neonatal h*TNF-*α tg mice. In brief, calvariae were digested in α-MEM medium (PAN Biotech, Aidenbach, Germany) with 0.1% collagenase type IA (Sigma-Aldrich) and 0.2% dispase II (Roche, Basle, Switzerland) at 37°C on a shaker for 5 × 10 min. Fractions 2–5 were collected and cells were cultured and expanded until P2 to P3. At subconfluency state, cells were plated at 104/cells/cm^2^. Mineralization assays were carried out in 12-well plate by changing the medium to osteoblast mineralization medium (PromoCell, Heidelberg, Germany) at 100% confluency and cells were irradiated 24 h after the first medium change. Mineralization media was used according to the manufacturer’s recommendation and formation of bone nodules was evaluated at d21 using Alizarin red stain (Millipore, Darmstadt, Germany). Total wells were scanned and images were then analyzed using ImageJ software (Version 1.46r).

### Enzyme-Linked Immunosorbent Assay

For *in vitro* experiments supernatants of the cell cultures were taken and, in the case of FLS, levels of IL-6, hTNF-α (Biolegend, San Diego, CA, USA), and KC (R&D, Minneapolis, MN, USA) were measured according to the manufacturer’s instructions. Samples were diluted 1:5 prior to the measurement. Supernatants of osteoblast cultures were taken accordingly, diluted 1:100 and OPG levels were determined using an R&D ELISA according to the manufacturer’s instructions.

### RNA and Quantitative PCR

Total RNA from cell culture was isolated using TriFast (peqlab, Darmstadt, Germany) and phenol–chloroform extraction. 0.8 µg of total RNA was transcribed into cDNA using the QuantiTect^®^ reverse transcription kit by Quiagen (Hilden, Germany) according to the manufacturer’s recommendations. Quantitative real-time PCR was carried out using SYBR Green (Thermo Scientific, Waltham, MA, USA) as fluorescent dye. Genes of interest were normalized to at least two housekeepers. Target stability values for these housekeepers were tested and a coefficient variance of <0.5 was accepted as stable. Obtained data were analyzed using the CFX Manager 3.1 software (BioRad, Hercules, CA, USA). Primes were either designed using Primer Blast (NCBI) and manufactured by MWG (Eurofins Genomics GmbH, Ebersfeld, Germany) or by using BioRad PrimePCR products (BioRad Laboratories, Inc., Hercules, CA, USA), respectively. Efficiency of primers was either provided by the manufacturer or determined prior to use. Table 1 provides an overview of the used primers with their sequences or, in case of commercially obtained primers, unique assay IDs.

**Table 1:** List of self-designed and commercially obtained primers with the respective sequences or assay IDs.

MWG primers		

Symbol	Forward [5′→3′]	Reverse [3′→5′]
Acp5	CGACAAGAGGTTCCAGGAGA	TTCCAGCCAGCACATACCAG
ACTB	ACAGCTTCTTTGCAGCTCCTTCG	ATCGTCATCCATGGCGAACTGG
B2M	CTGCTACGTAACACAGTTCCACCC	CATGATGCTTGATCACATGTCTCG
catK	GGC CAG TGT GGT TCC TGT T	CAG TGG TCA TAT AGC CGC CTC
Dentin matrix protein-1	GTG CCC AAG ATA CCC CCA G	GCA TCC CTT CAT CAT CGA ACT CA
GAPDH	AGGTCGGTGTGAACGGATTTG	GGGGTCGTTGATGGCAACA
HMBS	GAGTCTAGATGGCTCAGATAGCATGC	CCTACAGACCAGTTAGCGCACATC
HPRT	GTTGGGCTTACCTCACTGCTTTC	CCTGGTTCATCATCGCTAATCACG
MMP-3	GCTGTCTTTGAAGCATTTGGGTT	ACAATTAAACCAGCTATTGCTCTTC
omd	GTGAGCAGAGGAGTACTAACGG	TGACTGTCATGGTCGTCTTCC
Tnfsf11/RANK-L	ACC AGC ATC AAA ATC CCA AG	TTT GAA AGC CCC AAA GTA CG
sparc	CCTTCGACTCTTCCTGCCAC	GCGATGTATTTGCATGGTCCG
TBP	TCTGAGAGCTCTGGAATTGTACCG	TGATGACTGCAGCAAATCGCTTG

**BioRad primers**		

**Symbol**	**Unique assay ID**	

CXCL1	qMmuCED0047655	
hTNF-α	qHsaCED0037461	
MMP-9	qMmuCID0021296	
MMP-13	qMmuCID0025884	
RPS18	qMmuCED0045430	
TGFβ1	qMmuCID0017320	
Tnfrsf11b/OPG	qMmuCID0027158	

### Analyses by Flow Cytometry

For flow cytometry analyses, cells were trypsinized and, in the case of cell death analysis, resuspended in ringer’s solution (Braun, Melsungen, Germany) containing 0.2 µg/ml AnnexinV-FITC (AxV) and 0.4 µg/ml propidium iodide (PI) and consecutively stained for 30 min at 4°C in the dark. Cells were then measured with an EPICS XL-MCL (Beckman Coulter, Brea, CA, USA) flow cytometer and evaluated with the Kaluza Analysis software (Beckman Coulter). AxV−/PI− cells were considered as live cells, while AxV+/PI− and AxV+/PI+ cells were considered to be apoptotic and necrotic, respectively. In the case of surface staining of FLS, 1 × 106 cells were resuspended in 100 µl 2% FBS/PBS and incubated at 4°C with saturated fluorochrome-labeled antibodies (BD Bioscience, Franklin Lakes, NJ, USA and eBiosciences, Frankfurt) for 30 min in the dark. Measurements were carried out with a Gallios flow cytometer (Beckman Coulter). For phenotyping of FLS cultures the following antibodies were used: CD11b-FITC (BD #557396), CD54-PE (eBioscience #12-0541), CD90.2-PE (eBioscience #12-0903), and CD106-FITC (eBioscience #11-1061). Evaluation of the samples was done with the Kaluza Analysis software.

### Statistical Analysis

Statistical analysis was performed by using GraphPad Prism software (GraphPad software, Inc., San Diego, CA, USA). All data are presented as mean ± SEM, tested for normal distribution and variance equality. Depending on whether data was normal distributed or not, either two-tailed *t*-test or non-parametric two-tailed Mann–Whitney *U* test was used for analyzation in comparison to mock-treated controls. Significances are indicated as follows: *p < 0.05, **p < 0.01, and ***p < 0.001.

## Results

### LD-RT Diminishes the Pro-Inflammatory Phenotype of FLS

**Figure 1:**
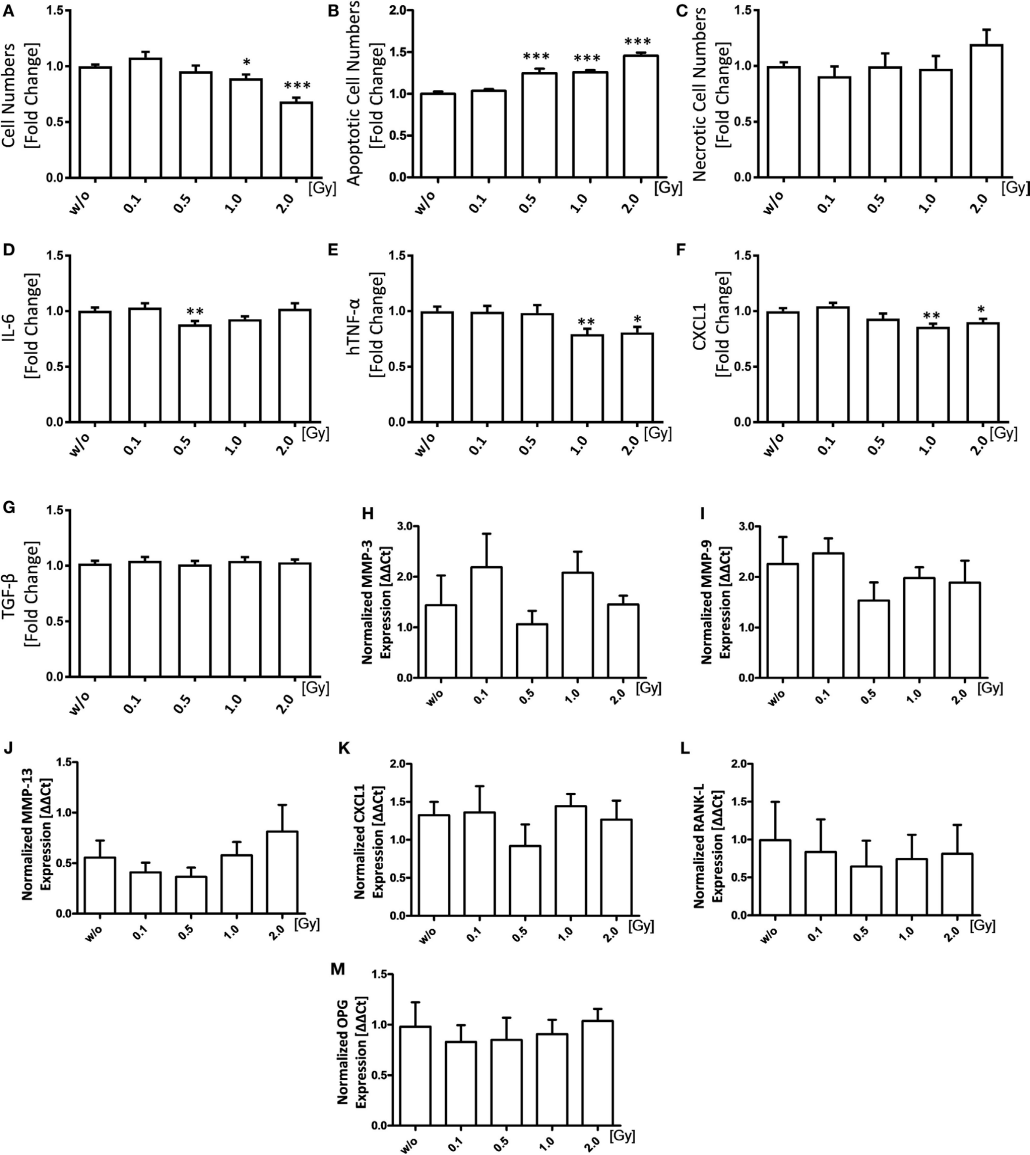
Low-dose irradiation induces apoptosis of fibroblast-like synoviocytes (FLS) and slightly impacts on the inflammatory FLS phenotype. Inflammatory FLS obtained from h*TNF-*α tg mice were analyzed 96 h after irradiation with various doses of X-rays. Cell numbers **(A)** of living cells were counted using a Neubauer chamber and cell death was determined *via* flow cytometry analysis after staining with AxV-FITC/PI. Vital cells were defined as AxV^−^/PI^−^, apoptotic cells as AxV^+^/PI^−^ **(B)**, and necrotic ones as AxV^+^/PI^+^ **(C)**. Quantification of secreted IL-6 **(D)**, hTNF-α **(E)**, CXCL1 **(F)**, and TGF-β **(G)** was performed 96 h after irradiation in supernatants of FLS cultures *via* enzyme-linked immunosorbent assays. Total RNA levels were isolated using phenol–chloroform extraction. Gene expression was analyzed using SYBR Green quantitative PCR analysis **(H–M)**. Depicted is joint data obtained of five h*TNF-*α tg-FLS cell lines, examined in four independent experiments, each performed at least in triplicates. Data are presented as mean ± SEM, tested for normal distribution, and analyzed by students t test **(A–G)** or two-tailed Mann–Whitney *U* test **(H–M)** in comparison to mock-treated (w/o) controls at 96 h after irradiation (*p < 0.05, **p < 0.01, and ***p < 0.001).

As FLS demonstrate a proliferative pro-inflammatory phenotype, we first examined the impact of LD-RT on these characteristics: irradiation of FLS with a single dose of 1.0 and 2.0 Gy reduced FLS cell numbers significantly (Figure 1A). Additionally, a single dose ranging from 0.5 to 2.0 Gy highly significantly increased FLS apoptosis, while the number of necrotic cells remained stable (Figures 1B,C). We then investigated the impact of LD-RT on secreted levels of pro-inflammatory cytokines as well as cartilage-degrading proteins. The level of pro-inflammatory IL-6 was significantly reduced only after irradiation with 0.5 Gy, but not anymore after 1.0 or 2.0 Gy. In contrast, CXCL1 and human TNF-α (hTNF-α) were significantly reduced after irradiation with 1.0 or 2.0 Gy, while TGF-β levels remained stable (Figures 1D,G). One has, however, to mention that these significant, but slight LD-RT-induced reductions alone might not have biological significance and that rather osteoclastogenesis is influenced by LD-RT.

qPCR analyses of expression levels of various enzymes 96 h after irradiation revealed a slight reduction of mRNA levels of cartilage-degrading MMPs, pro-inflammatory *CXCL1*, and *receptor activator of nuclear factor* κ*B-ligand* (*RANK-L*) after irradiation with again 0.5 Gy (Figures 1H–L), with only marginal changes in the expression patterns of *osteoprotegerin* (*OPG*) (Figure 1M). Even though secreted TGF-β protein levels showed no significant alterations (Figure 1G), a tendency of increased mRNA TGF-β expression following irradiation with a single dose of 0.5 Gy (Figure S1 in Supplementary Material) was observed. In contrast, the ΔΔCt values of hTNF-α showed a slight decrease after radiation exposure (Figure S1 in Supplementary Material).

### LD-RT Reduces the Numbers of Differentiating Osteoclasts

**Figure 2:**
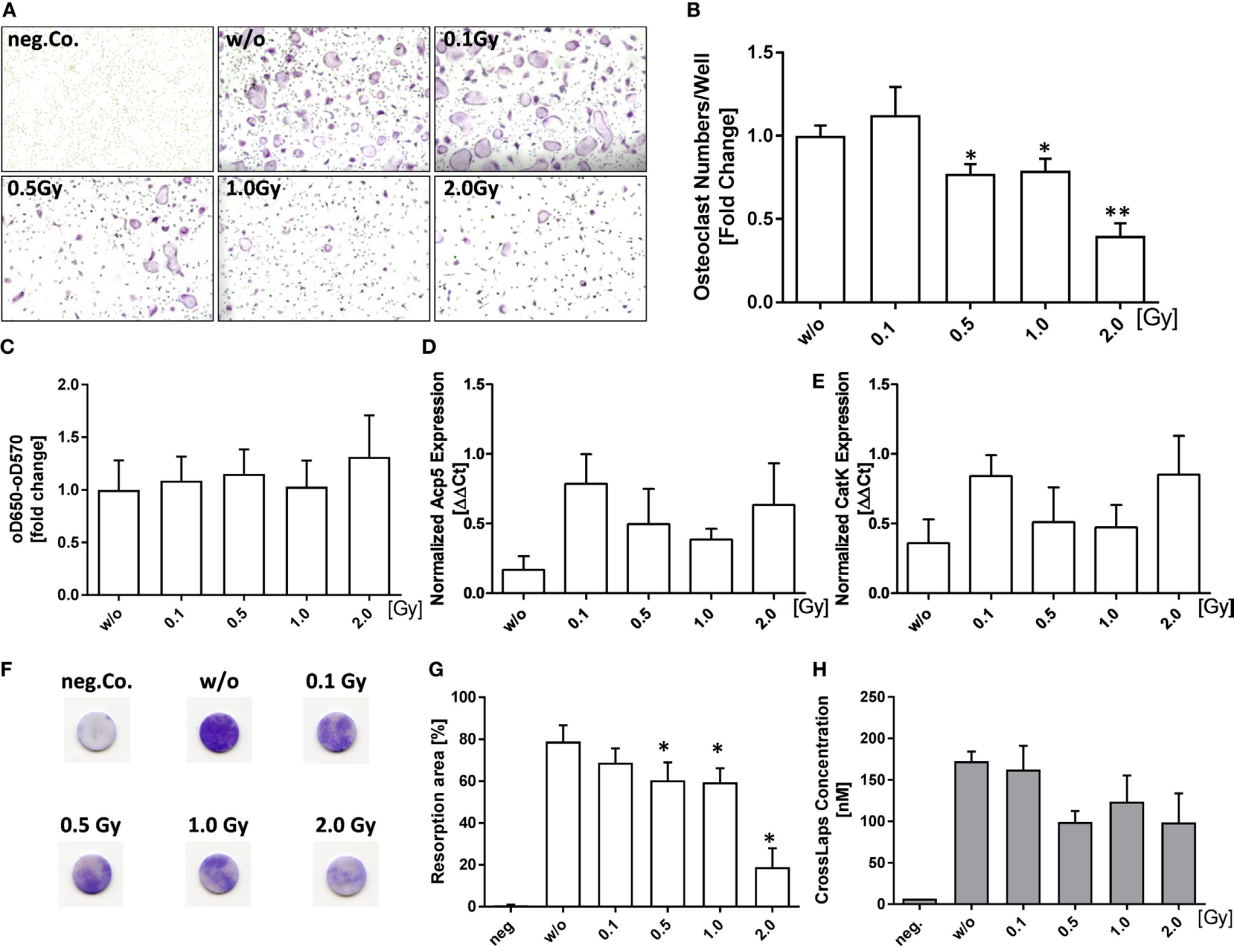
Low-dose irradiation reduces the numbers and activity of differentiated inflammatory osteoclasts. Differentiation of h*TNF-*α tg bone marrow cells into osteoclasts (OCs) was carried out in the presence of 10 ng/ml M-CSF and 50 ng/ml RANK-L. Cells were irradiated with the depicted doses of X-rays 24 h after seeding. Differentiated cells were stained for tartrate-resistant acid phosphatase (TRAP). Representative images where taken at 5× magnification **(A)**. OC numbers of three fields/well, taken at 5× magnification, were counted and OC numbers of each well were pooled. TRAP^+^ cells with three or more nuclei were considered to be OCs **(B)**. Cell viability was analyzed by MTT assay **(C)**. Total mRNA from differentiated OCs was isolated using phenol–chloroform extraction and quantitative real-time PCR (qPCR) for *Acp5* and *CatK* using SYBR Green was carried out **(D,E)**. Depicted is joint data from differentiated pooled bone marrow of total 7 h*TNF-*α tg mice investigated in three independent experiments, each carried out in triplicates. For qPCR analysis mRNA triplicates of each experiment were pooled before qPCR analysis. Pit-formation assay was used to determine functionality of differentiated osteoclasts. Resorbing activity of osteoclasts was determined using toluidine blue staining of bone slices **(F,G)** as well as CrossLaps enzyme-linked immunosorbent assay [**(H)**, conducted on d7 of osteoclast/bone slice cultivation]. For MTT analysis, joint data from 9 h*TNF-*α tg mice carried out in three independent experiments in triplicates is depicted. Pit-formation assay is represented by the pooled data of four mice in two independent experiments, both performed in triplicates. Data are presented as mean ± SEM and analyzed by two-tailed Mann–Whitney *U* test in comparison to mock-treated controls (w/o) (*p < 0.05 and **p < 0.01).

As RA is characterized by a misbalance in bone homeostasis represented by OC-mediated bone loss, we investigated if LD-RT impacts on osteoclastogenesis. Bone marrow cells of h*TNF-*α tg mice were irradiated 24 h after culture containing M-CSF and RANK-L. A single dose of 0.5 Gy or more significantly reduced differentiated OCs numbers as verified by TRAP stain (Figures 2A,B). Of note is that this was not due to reduced viability as determined by MTT assay (Figure 2C). Interestingly, mRNA levels of osteoclast enzymes [*cathepsinK* (*catK*) and *acid phosphatase 5, tartrate resistant* (*Acp5*)] were slightly elevated after irradiation. Doses of 0.5 and 1.0 Gy resulted in a slight reduced expression of these enzymes as compared to those after irradiation with 0.1 or 2.0 Gy; however, levels remained above the mock-treated controls (Figures 2D,E). The analyses of the activity of osteoclasts revealed a significant reduced pit formation (Figures 2F,G) and slightly reduced Cross Laps concentration (Figure 2H) after irradiation with 0.5 Gy and higher.

### LD-RT Increases Mineralization Properties of Osteoblasts

**Figure 3:**
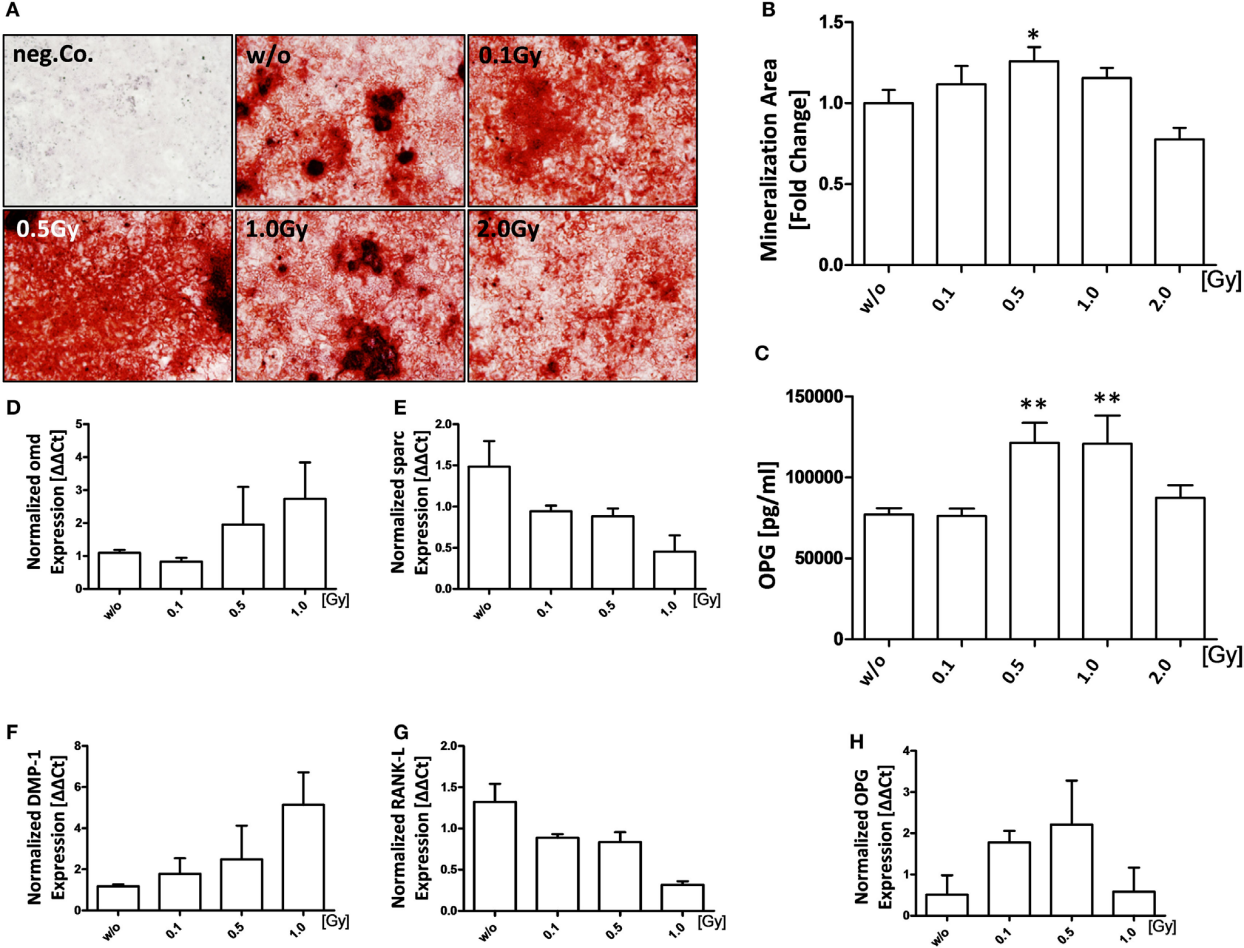
Low-dose irradiation increases mineralization properties of osteoblasts (OBs). Pre-osteoblasts (pOBs) were isolated from the calvariae from 3–6 days old neonatal h*TNF-*α tg mice. Cells were seeded and irradiated at d1 after the addition of mineralization media that was added after cells reached about 80–90% confluency. **(A)** Shows Alizarin red stains at d21; representative images were taken at 5× magnification. Mineralization areas of full wells were quantified using ImageJ **(B)**. Supernatants from d7 were analyzed for OPG *via* enzyme-linked immunosorbent assays **(C)**. Total mRNA from d21 was isolated, the triplicates of each cell line were pooled and quantitative real-time PCR for selected OB markers was carried out **(D–H)**. Depicted is joint data from two independent experiments with three independently generated OB lines. Each experiment was carried out in triplicates. Data are presented as mean ± SEM and analyzed by two-tailed Mann–Whitney *U* test in comparison to mock-treated controls (w/o) (*p < 0.05 and **p < 0.01).

Since bone destruction is tightly regulated by both osteoclasts and osteoblasts, we then examined if LD-RT has also an influence on bone formation by osteoblasts. For this, pre-osteoblasts were seeded in mineralization media, treated with LD-RT, and checked for bone nodules using Alizarin Red stain 21 days after irradiation (Figures 3A,B). A significant increase in mineralized area was only observed after irradiation with a dose of 0.5 Gy. In accordance with this finding, an elevated concentration of *OPG* was observed after exposure of osteoblasts to 0.5 or 1.0 Gy (Figure 3C). The mRNA expression of osteomodulin (*omd*), dentin matrix protein-1 (*DMP-1*), and OPG was also slightly, but not significantly increased after irradiation of osteoblasts with 0.5 Gy (Figures 3D,F,H). In contrast, levels of osteonectin (*sparc*) and *RANK-L* were decreased after irradiation (Figures 3E,G). An increase of the gene expression ratio of OPG/RANK-L at 0.1 and 0.5 Gy was present.

### Establishment of an *In Vivo* Irradiation Set-Up

**Figure 4:**
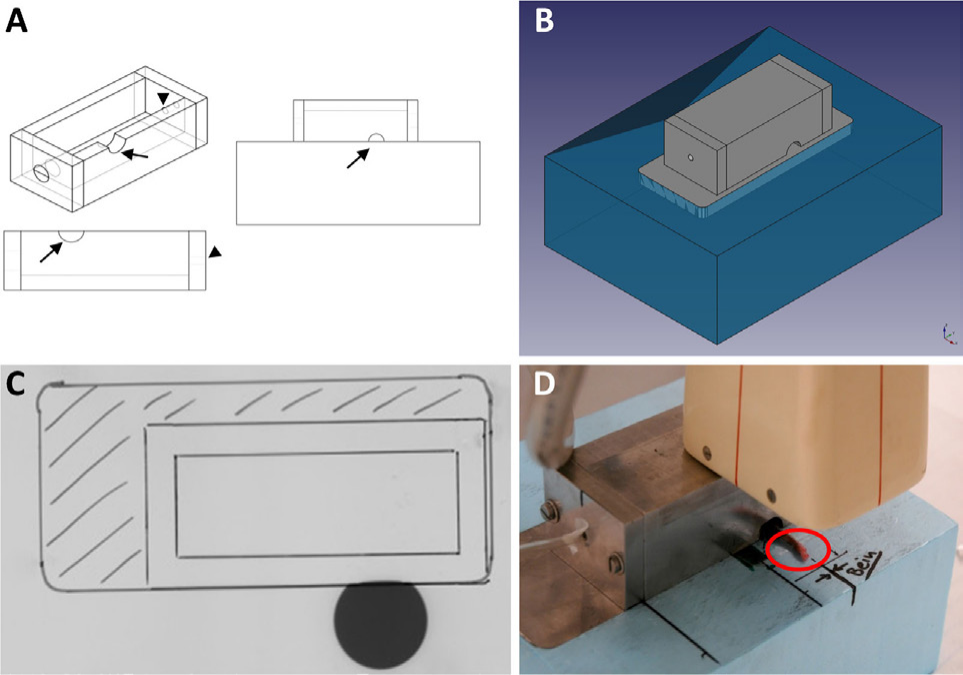
Establishment of a local *in vivo* irradiation procedure for hind limbs of h*TNF-*α tg mice. The applicator **(A)** is built from 10 mm lead pieces in order to shield the mouse from irradiation. Only the left hind leg can be pulled through an opening (indicated by arrows). Another opening (indicated by arrowheads) allows for a tube, releasing anesthetics into the chamber, to be inserted. This ensures that the mice stay into place during the whole procedure. The whole set-up then gets placed onto a 10 mm lead plate that is embedded into an 80 mm Styrodur^®^ block **(B)**. The embedded lead plate guarantees that the mouse is completely protected from X-rays and only the exposed limbs receive the intended dose of 0.5 Gy. Image **(C)** shows proof-of-concept on radiographic film: the gray circle indicates that only the exposed limb is receiving a dosage. The body of the mouse is efficiently shielded from X-rays by the lead applicator. The placement of the applicator and the embedded lead plate are indicated by markings on the film. Figure **(D)** shows a representative image of an irradiation procedure: the paw of the mouse is aligned according to pre-made markings on the Styrodur^®^ block, allowing for a precise and reproducible local irradiation of only the exposed extremities. Irradiation is applied through an X-ray tube equipped with a 5 mm copper filter. Subsequently, a single dose of 0.5 Gy (250 kV, 15 mA) is applied.

As we aimed to examine the effects of LD-RT on inflammation and bone metabolism not only *in vitro*, but also *in vivo*, we succeeded to develop an irradiation set-up for mice that allows for a precise, fast, and reproducible irradiation procedure of the affected joints. In accordance with clinical applications, we established a set-up that allows a local irradiation of involved joints. Thus, a lead chamber was constructed on top of a Styrodur^®^ block with openings for anesthesia and the left hind leg of a mouse (Figures 4A,B) allowing local irradiation of only the exposed limb. Field of irradiation was verified on radiographic film (Figure 4C). An exemplary local irradiation set-up of a mouse is shown in Figure 4D.

### LD-RT Has a Positive Impact on Course of Disease in h*TNF-*α tg Mice

**Figure 5:**
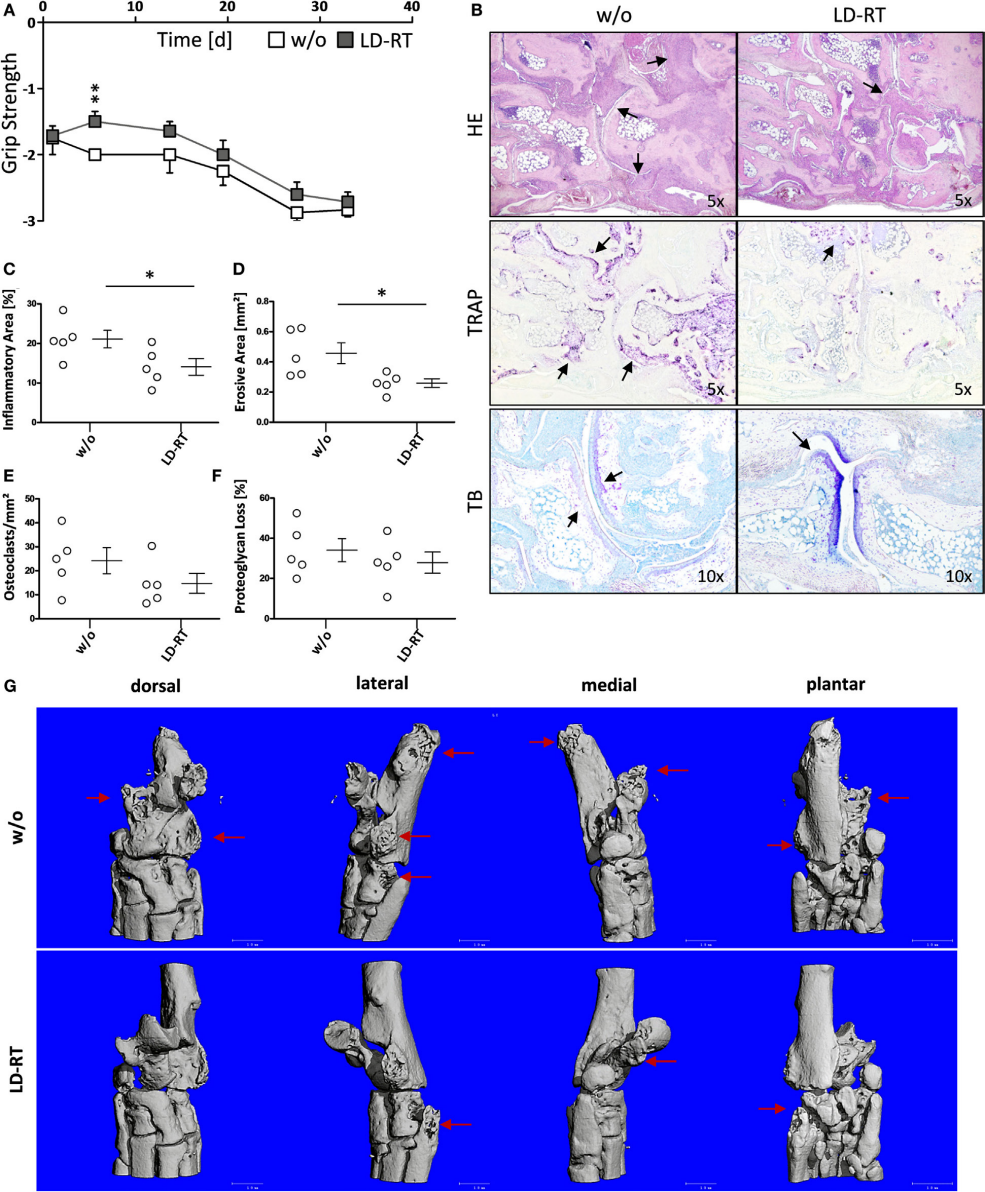
Low-dose irradiation increases grip strength and improves disease in h*TNF-*α tg mice. 8-week-old h*TNF-*α tg mice suffering from established polyarthritis (PA) were locally irradiated with 0.5 Gy of X-rays. Sham-irradiated (w/o) mice served as control animals. Mice were regularly scored for grip strength in the treated leg **(A)** in a blinded manner. 33 days after irradiation mice were sacrificed and histomorphological analyses of the left hind paw was carried out. **(B)** Shows representative histology images of the tarsal joints stained with hematoxylin and eosin (H&E), tartrate-resistant acid phosphatase (TRAP), and toluidine blue (TB), taken at 5 and 10× magnification, respectively as indicated. Arrows point to inflamed tissue (H&E), erosive areas, and TRAP positive cells (TRAP) as well as unstained cartilage next to stained proteoglycan (TB). **(C–F)** Shows histomorphometric analyses of hind paws: **(C)** inflammatory area, **(D)** erosive areas of the bone and numbers of TRAP positive cells [osteoclasts, **(E)**], as well as the percentage of unstained proteoglycan **(F)**. Histomorphological analyses were carried out using the OsteoMeasure™ Software. Data show three independent experiments with in sum n_w/o_ = 6 and n_local_ = 5 h*TNF-*α tg mice; mice were age- and sex-matched. Data are presented as mean ± SEM and analyzed by two-tailed Mann–Whitney *U* test in comparison to mock-treated (w/o) controls (*p < 0.05). **(G)** Shows exemplarily micro CT images from one control (w/o) and one low-dose radiotherapy treated animal, respectively. Arrows mark the erosive areas.

**Figure 6:**
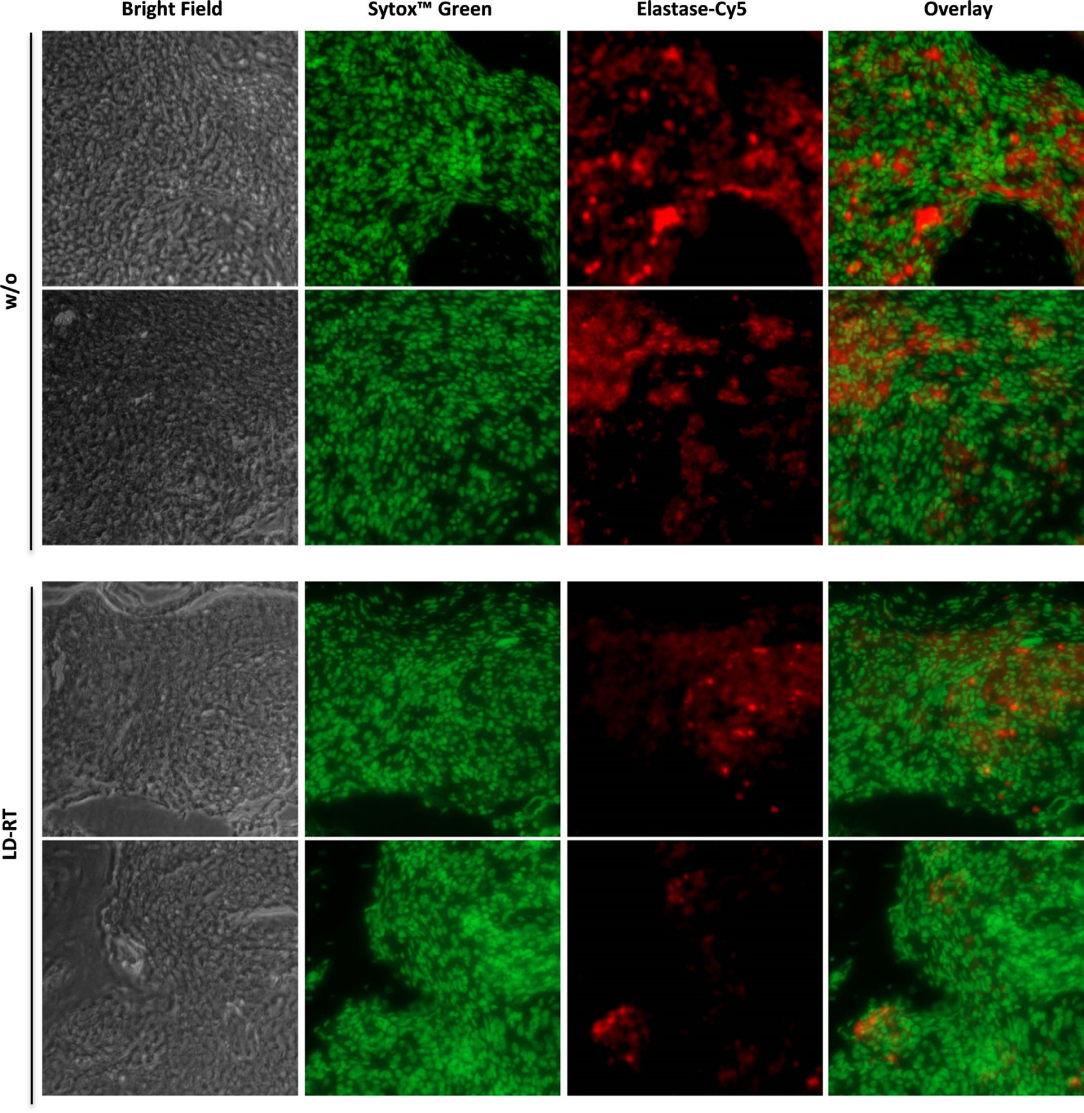
Low-dose irradiation impacts on neutrophil infiltration and neutrophil extracellular trap formation. 8-week-old h*TNF-*α tg mice suffering from established polyarthritis (PA) were locally irradiated with 0.5 Gy of X-rays. Sham-irradiated (w/o) mice served as control animals. 33 days post-irradiation, animals were sacrificed and hind paws were decalcified and embedded in paraffin. Sections of embedded paws were stained for elastase-Cy5 and sytox™ green. Pictures were randomly taken at 20× magnification using a Zeiss microscope. Exemplary images from two mice per treatment are depicted.

h*TNF-*α tg mice (strain tg197) received irradiation of the left hind limb with a single dose of 0.5 Gy—the lowest dose that was most beneficial *in vitro*. Clinical parameters and symptoms in LD-RT and mock-treated tarsal joints were closely monitored over a time period of 33 days. Grip strength in paws treated with 0.5 Gy was significantly maintained during the first 7 days after LD-RT and remained slightly improved compared to mock-treated (w/o) animals during the entire observation period (Figure 5A). Histomorphometry (Figure 5B) of the tarsal joints was performed and H&E sections of the hind paws revealed significant reduction of inflammatory areas (Figure 5C) in LD-RT-treated animals. Further, TRAP stain showed a significant decrease of osteoclast-mediated erosive areas (Figure 5D), alongside with reduced osteoclast numbers (Figure 5E). Toluidine blue staining revealed a slight reduction in cartilage loss following LD-RT of the hint limb (Figure 5F). Micro CT images (Figure 5G) further verified the positive effects of LD-RT on the bone. Further, Paraffin-embedded sections of the left hind paw of irradiated and control animals were stained for elastase in order to take a closer look at neutrophil infiltration and NET formation. In irradiated joints a reduced number of neutrophils with slightly reduced NET formation were observed (Figure 6). In addition, histomorphometry of additionally of the LD-RT protected leg of irradiated mice showed slightly reduced inflammatory infiltrates in both treated and protected leg, while a significant reduction of erosive areas was only found in the LD-RT-treated leg (Figure S2 in Supplementary Material).

## Discussion

Despite the success of anti-rheumatic drug therapy a substantial proportion of RA patients fail to achieve remission (41). Novel and/or complementary treatments would, therefore, be of value (42). Our results suggest that LD-RT with particularly a single dose of 0.5 Gy might be a useful add-on treatment for RA. We here show for the first time that LD-RT has a positive effect on FLS, OCs, and osteoblasts which are all involved in inflammation and degenerative processes in the joints. FLS show unique features such as hyperproliferation (9) and resistance to apoptosis (43). By treating h*TNF-*α tg FLS with various doses of radiation we found that single doses of 0.5–2.0 Gy are able to reduce proliferation. In particular with doses starting from 0.5 Gy a significant increase of apoptosis of FLS was observed while numbers of necrotic FLS remained stable. Since necrotic cells are rather inflammatory and apoptotic cells are anti-inflammatory (44, 45), LD-RT might ameliorate inflammation by apoptosis induction of FLS. However, as total apoptosis levels only rose by roughly 3% (untreated 6.78 ± 0.9%; 2.0 Gy 9.71 ± 1.02%) we exclude a pure cell death-related effect of LD-RT on FLS.

Further, FLS actively contribute to joint destruction by secreting pro-inflammatory cytokines as well as destructive enzymes (1, 4, 6, 46). Treatment of FLS with 0.5 Gy of X-rays resulted in a significant, but only slight reduction of IL-6 in a discontinuous dose dependency. Such dose dependencies are often observed in immune and endothelial cells in the dose ranges of LD-RT (47) and we here demonstrate for the first time that they also appear after exposure of FLS to LD-RT. This was also seen for the mRNA expression of *MMP-3/-9/13* and *CXCL1* (Figure 1). Further, LD-RT had a counter-rotating effect on pro- and anti-inflammatory cytokines in FLS (Figure S1 in Supplementary Material) with an increase of TGF-β following radiation with 0.5 Gy and slight decreased expression of TNF-α after radiation exposure in general. On protein level, only TNF-α was significantly decreased after exposure to 1.0 and 2.0 Gy, as it was the case for CXCL1. Also, there is evidence that degradation of non-collagen matrix components of the joints can be reduced by irradiation with 0.5 Gy as a trend of lowered expression of MMPs was observed. In RA, collagenases, such as MMP-13, are able to degrade otherwise intact fibrillary collagen and thus compromise cartilage strength (48). A reduced expression of MMP-13 might, therefore, lead to increased cartilage stability.

As pro-inflammatory cytokines are associated with pain (49), even a slight reduction of IL-6 and also that of hTNF-α levels might contribute to the reduced pain levels that patients experience after LD-RT (50). Furthermore, decreased levels of hTNF-α have been shown to contribute to a reduction in OC numbers (51, 52) and to an increase in osteoblast function (53). As immune activation and bone loss are linked to each other (54) and bone erosion in RA is often associated with inflammation, both occur within close proximity in the synovium (55).

As elevated numbers and activity levels of OCs are responsible for bone damage in RA (56), we examined the impact of radiation on OC differentiation and function. Doses from 0.5–2.0 Gy significantly reduced numbers of differentiated OCs, but did not impact on their viability, indicating that cell death is not responsible for reduced OCs numbers after LD-RT. The analyses of functional markers of bone resorption such as *Acp5* and c*atK* (57–59) revealed that expression levels of *Acp5* and c*atK* were highest after irradiation with 0.1 or 2.0 Gy. This again highlights that discontinuous dose-effects in the low-dose range have to be carefully considered when introducing LD-RT for RA in the clinic (26, 29, 60). Expression levels were lowest after irradiation with doses of 0.5 and 1.0 Gy. It has to be stressed that these mRNA expression levels were analyzed at the end of the differentiation process. mRNA levels of *Acp5* and c*atK* might be upregulated because of an impaired osteoclast function, since the OCs then try to compensate it by up-regulating of lytic enzymes. When looking more closely at the OC activity we revealed that the Cross Laps concentration was lowest after irradiation with 0.5 Gy and that pit formation was significantly reduced at doses of 0.5 Gy and higher (Figures 2F,H).

It has already been shown that low-dose radiation can have a positive effect on normal bone formation through increased osteoblast activity and numbers (35). We now demonstrated for the first time that this also applies in an inflammatory state. Next to a significant increase in mineralized areas only after LD-RT with a dose of 0.5 Gy, a significant increase in OPG protein levels was seen after irradiation with 0.5 or 1.0 Gy. These data confirm discontinuous dose dependencies also for mineralization of osteoblasts following LD-RT. Further, a trend of reduced mRNA expression of *RANK-L* was observed after LD-RT.

Further, the expression of *Omd* and *DMP-1* slightly increases at 0.5 and 1 Gy while the mineralization is significantly increased only after 0.5 Gy. *Omd* is a maturation marker that is upregulated during maturation of osteoblasts; it is secreted into the matrix and is a marker for mineralized tissue (61, 62); after 1.0 Gy we still have mineralized tissue. *DMP-1* seems to be of importance during osteoblastogenesis and was found to be expressed in the nucleus of differentiated OBs (63). Therefore, this marker is not directly connected to mineralization which is detected by Alizarin red stain. The latter is used to visualize calcium deposition in mineralized matrix at the end of the mineralization process. Therefore, the pattern of expression should differ. Nevertheless, as these factors are involved in osteoblast differentiation and maturation as well as bone formation, their increased expression indicates a positive effect of LD-RT on bone formation (61, 63).

To examine the impact of locally applied LD-RT *in vivo*, we first had to set up a mouse irradiation procedure that closely resembles the clinical situation (Figure 4). As doses of 0.5 Gy were most effective in the *in vitro* model systems as well as in previously published work on anti-inflammatory effects of LD-RT (16, 47, 64), we locally irradiated the left hind limb of h*TNF-*α tg mice with 0.5 Gy. An increase in grip strength was observed following LD-RT and was used for disease assessment: since h*TNF*α-tg mice produce continuously TNF-alpha swelling is a less optimal factor for assessment of disease in these mice. In contrast, the ability to hold onto a wire remained stable. Further, h*TNF*α tg mice show less swelling in comparison to that observed with other model such as the serum transfer model or in collagen-induced arthritis. In addition, patients often report an increase in swelling, but an improvement in pain perception and functionality in irradiated joints after LD-RT. Detailed histomorphometric analyses further revealed a reduced inflammatory area identified by infiltrating immune cells as well as a reduced erosive area with less OCs (Figure 5). As neutrophils are the most abundant circulating leukocytes and are key immune cells that contribute to the maintenance of inflammation in autoimmune diseases (10, 11), we additionally analyzed neutrophils and NET formation in the joints and revealed a reduction after LD-RT (Figure 6).

These *in vivo* data are in accordance with the *in vitro* results and illustrate that LD-RT is capable of slowing down disease progression in advanced RA. Even though a tendency of reduced inflammatory area was observed in both, the irradiated and in the non-irradiated leg of the mice, suggesting systemic immune-mediated effects, these changes were not significant. We suggest that LD-RT particularly locally impacts on bone metabolism, since reduced bone erosion was only found in the irradiated leg (Figure S2 in Supplementary Material).

**Figure 7:**
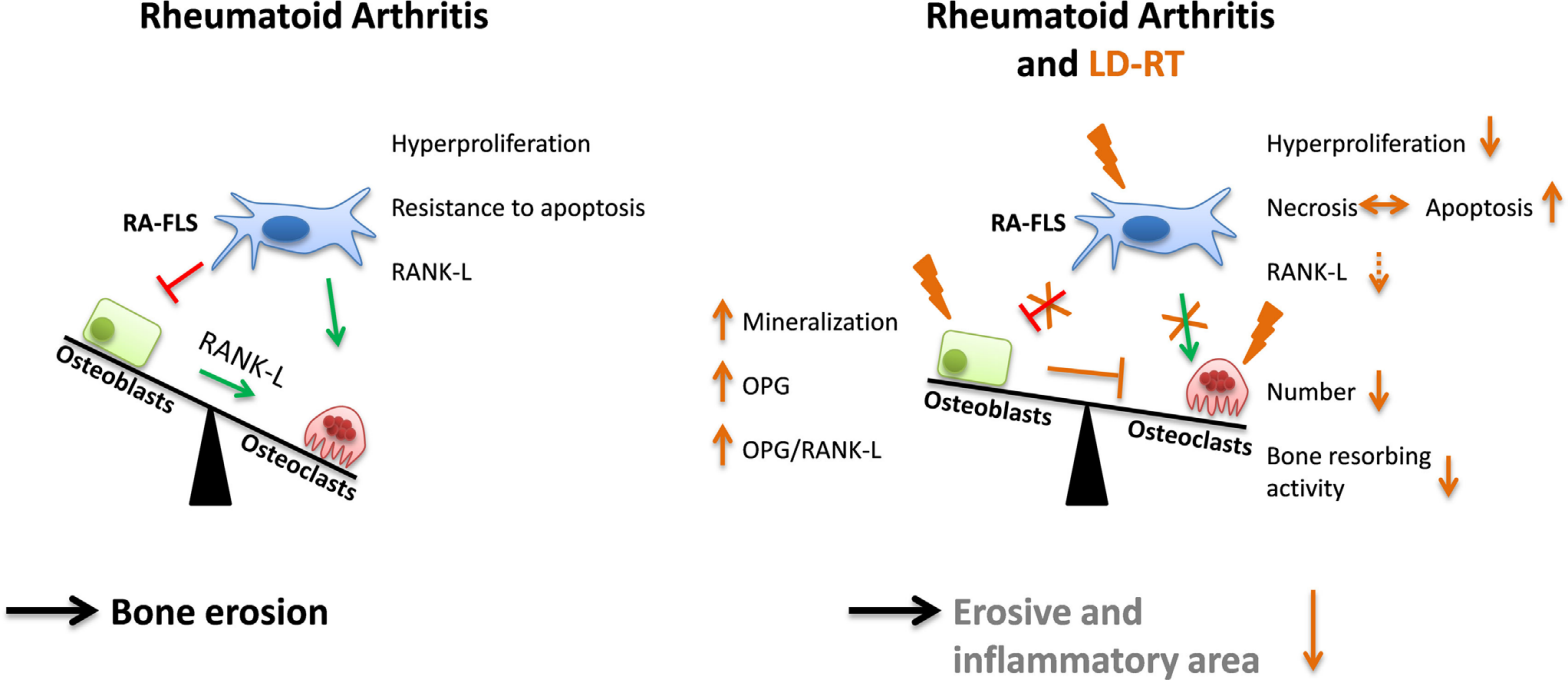
Low-dose irradiation positively impacts on bone metabolism. In rheumatoid arthritis (RA) a misbalanced relationship of bone resorption by osteoclasts and bone formation by osteoblasts leads to destruction of joints. In the affected joint, fibroblasts like synoviocytes (RA-FLS) show a rather aggressive phenotype that is characterized by hyperproliferation, resistance to apoptosis, and receptor activator of nuclear factor κB-ligand (RANK-L) expression. Following low-dose radiotherapy (LD-RT) with in particular 0.5 Gy, the inflammatory (reduced neutrophil extracellular trap) and erosive area is reduced, since LD-RT particularly positively impacts on RA-FLS, osteoblasts, and osteoclasts as schematically displayed in the illustration. Abbreviation: OPG, osteoprotegerin.

These results suggest that there are multiple LD-RT-induced mechanisms that are reacting on and within different cells that are present in the affected joints. Our results indicate that LD-RT in particular with a dose of 0.5 Gy has a positive influence on bone homeostasis in established RA (Figure 7).

## Ethics Statement

Mice were maintained in a SPF facility under sterile atmosphere at the animal facility of the Universitätsklinikum Erlangen (Franz-Penzoldt-Center). The animal procedures have been approved by the “Regierung of Unterfranken” and were conducted in accordance with the guidelines of Federation of European Laboratory Animal Science Associations (FELASA).

## Author Contributions

UG and BF designed the experiments and contributed to the analysis of the data. UG substantially contributed to writing of the article. BF designed the *in vivo* irradiation chamber together with JW and WS and performed *in vivo* irradiation together with LD. LD contributed to the design of the experiments, performed the experiments, analyzed the data, and contributed to writing of the article. AD helped with the design of the experiments and contributed to the data analyses. AH and MH critically revised the article and contributed to the experimental design. GS helped with the design of the experiments and contributed to writing of the article. JW helped with the design of the *in vivo* radiation set-up and performed the technical drawings. JH performed the neutrophil analyses. P-FR and JF helped in the experiments. WS helped in the calibration of the radiation procedure of mice. RF contributed to the design of the work.

## Conflict of Interest Statement

The authors declare that the research was conducted in the absence of any commercial or financial relationships that could be construed as a potential conflict of interest. The reviewer IN and handling Editor declared their shared affiliation.

## Supplementary Material

Figure S1 Low-dose irradiation has a counter-rotating effect on pro-and anti-inflammatory cytokines in fibroblast-like synoviocytes (FLS). Inflammatory FLS obtained from h*TNF-*α tg mice were analyzed 48 h after irradiation with various doses of X-rays. Total RNA levels were isolated using phenol–chloroform extraction. Gene expression of TNF-α **(A)** and *TGF-*β **(B)** was analyzed using SYBR Green quantitative PCR Analysis. Depicted is joint data consisting of five h*TNF-*α tg-FLS cell lines, examined in four independent experiments, each performed in triplicates. Data are presented as mean ± SEM.

Figure S2 Local low-dose irradiation particularly locally impacts on bone metabolism in hTNF-α tg animals. In order to investigate local and systemic effects of low-dose radiotherapy, inflammatory and erosive areas in the protected, non-irradiated leg were compared to those of mock-treated (control) animals **(A)**. Hind paws of animals that have received 0.5 Gy of local irradiation (treated, 0.5 Gy) on the left hind limb were compared to the right hind limb of these animals (protected) as well as the left, untreated limb of the control (untreated) with respect to inflammatory **(B)** and erosive areas **(C)**. Data show three independent experiments with in sum five mice per group; mice were age- and sex-matched. Data are presented as mean ± SEM and analyzed by two-tailed Mann–Whitney *U* test in comparison to mock-treated (w/o) controls (**p* < 0.05).
