# Supplementary figures and images for: Low-Dose Radiotherapy Ameliorates Advanced Arthritis in hTNF-α tg Mice by Particularly Positively Impacting on Bone Metabolism
Source: Front Immunol. 2018 Sep 18;9:1834. doi: 10.3389/fimmu.2018.01834 (PMC6153886; doi:10.3389/fimmu.2018.01834)

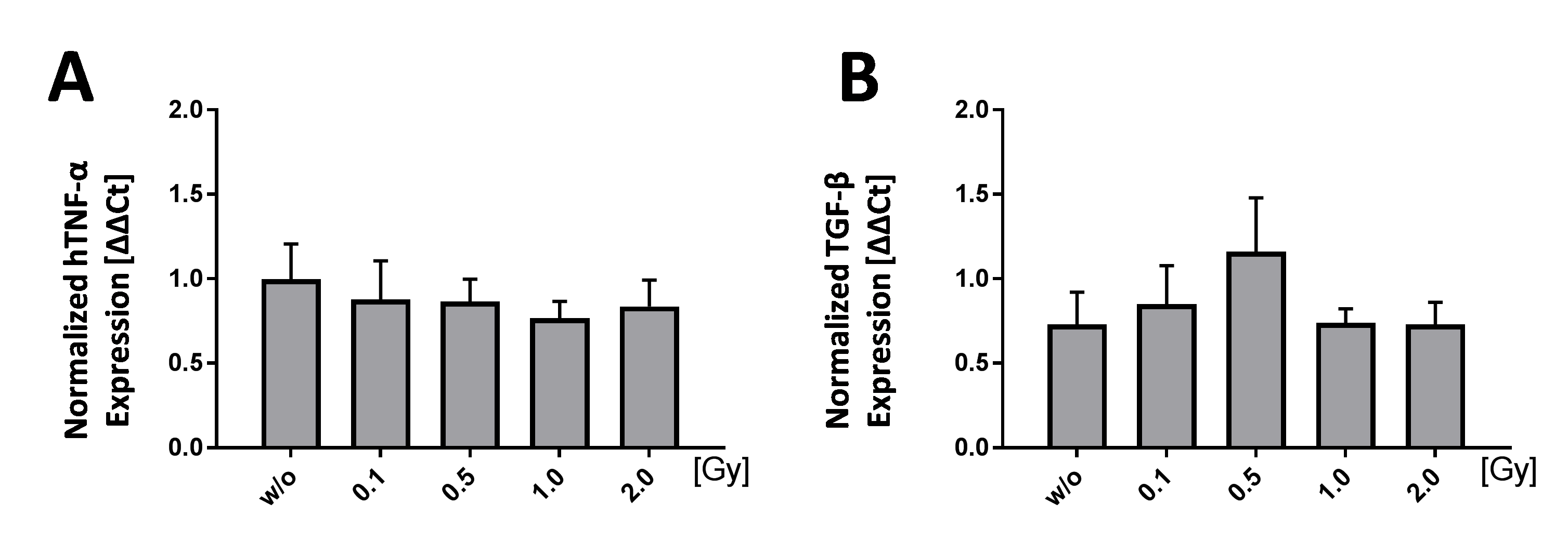

Supplement: Figure S1 — Low-dose irradiation has a counter-rotating effect on pro-and anti-inflammatory cytokines in fibroblast-like synoviocytes (FLS). Inflammatory FLS obtained from hTNF-α tg mice were analyzed 48 h after irradiation with various doses of X-rays. Total RNA levels were isolated using phenol–chloroform extraction. Gene expression of TNF-α (A) and TGF-β (B) was analyzed using SYBR Green quantitative PCR Analysis. Depicted is joint data consisting of five hTNF-α tg-FLS cell lines, examined in four independent experiments, each performed in triplicates. Data are presented as mean ± SEM. [file image_1.tif]

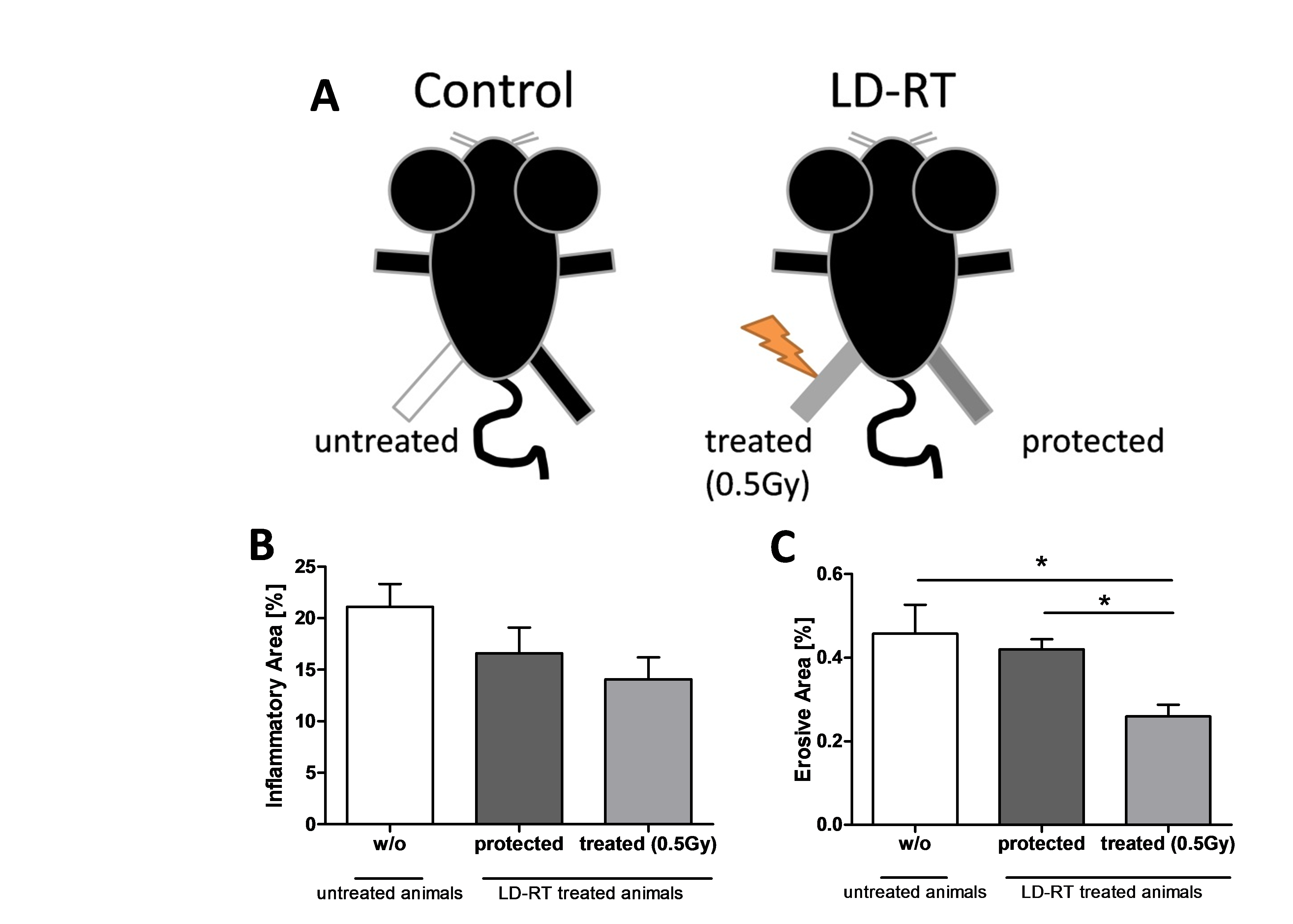

Supplement: Figure S2 — Local low-dose irradiation particularly locally impacts on bone metabolism in hTNF-α tg animals. In order to investigate local and systemic effects of low-dose radiotherapy, inflammatory and erosive areas in the protected, non-irradiated leg were compared to those of mock-treated (control) animals (A). Hind paws of animals that have received 0.5 Gy of local irradiation (treated, 0.5 Gy) on the left hind limb were compared to the right hind limb of these animals (protected) as well as the left, untreated limb of the control (untreated) with respect to inflammatory (B) and erosive areas (C). Data show three independent experiments with in sum five mice per group; mice were age- and sex-matched. Data are presented as mean ± SEM and analyzed by two-tailed Mann–Whitney U test in comparison to mock-treated (w/o) controls (*p < 0.05). [file image_2.tif]
